# The SUPPORT-S Protocol Study: A Postvention Program for Professionals After Patient or User Suicide

**DOI:** 10.3389/fpsyg.2020.00805

**Published:** 2020-05-05

**Authors:** Edouard Leaune, Bruno Cuvillier, Maxime Vieux, Michèle Pacaut-Troncin, Benoît Chalancon, Anne-Fleur Perez, Julie Haesebaert, Nicolas Chauliac, Emmanuel Poulet, Christine Durif-Bruckert

**Affiliations:** ^1^Center for Suicide Prevention, Centre Hospitalier le Vinatier, Bron, France; ^2^INSERM, U1028, CNRS, UMR 5292, Lyon Neuroscience Research Center, Psychiatric Disorders: from Resistance to Response – PSYR2 Team, Lyon, France; ^3^Groupe de Recherche en Psychologie Sociale, Lumière Lyon 2 University, Lyon, France; ^4^CNRS, UMR 5191, Interactions, Corpus, Apprentissages, Représentations, Ecole Normale Supérieure de Lyon, Lumière Lyon University, Lyon, France; ^5^Institut Régional Jean Bergeret, Centre Hospitalier Saint-Jean de Dieu, Lyon, France; ^6^EA 7425 HESPER Health Services and Performance Research – Claude Bernard Lyon 1 University, Lyon, France

**Keywords:** suicide, postvention, institution analysis, mixed method approach, action research

## Abstract

**Background:**

Exposure to patient or user suicide (PUS) is identified as a challenging occupational hazard for mental health and social work professionals. Professionals exposed to PUS may encounter several ranges of emotional, traumatic or professional impacts in the aftermath. A high proportion of exposed professionals reports a lack of support in the aftermath of PUS. SUPPORT is a postvention program designed to provide a comprehensive, adaptative and effective support to professionals impacted by PUS. The aims of the SUPPORT-S study are to (1) improve the design of the SUPPORT program, (2) evaluate the effectiveness of the program to buffer the emotional, traumatic and professional impacts and to improve the perceived social support for professionals exposed to PUS, and (3) provide more insights into the consequences of PUS on both professionals and organizations.

**Method:**

The SUPPORT-S study is a mixed method collaborative and participatory action research. The simultaneous and complementary collection and analysis of qualitative and quantitative data will offer an in-depth evaluation of the implementation and the effectiveness of the program. The qualitative evaluation includes: (a) an ethnographic observation; (b) 25 semi-directed interviews with randomized participants; (c) an activity analysis with providers of the program; and (d) collaborative sharing of the results with providers and participants. The quantitative evaluation includes pre- and post-measures in participants of: (a) emotional impact (*Differential Emotions Scale IV*); (b) traumatic impact (*Impact of Event Scale-Revised*); (c) professional impact (non-validated questionnaire); and (d) perceived social support (*Perceived Social Support Scale for Professionals*). The action research design will rely on: (a) the cycling process of implementation/evaluation/data sharing/adjustment and (b) the participatory approach through data sharing with providers and participants. Triangulation, saturation, randomization, and participatory design will also reduce the risk of biases and will improve the generalizability of conclusions.

**Expected Results:**

We expect the SUPPORT-S study to evaluate and improve the design of the SUPPORT program to effectively help professionals to cope with PUS.

**Conclusion:**

The results of the study will allow us to disseminate an effective and adaptive postvention program for professionals and institutions encountering PUS.

## IntroductIon

Exposure to patient or user suicide (PUS) has been identified as a frequent and challenging occupational hazard for mental health and social work professionals (Gulfi et al., 2010; Séguin et al., 2014; Castelli Dransart et al., 2017; Leaune et al., 2019a). For instance, between 51 and 82% of psychiatrists (Castelli Dransart et al., 2017), 46.9% of psychiatric trainees (Leaune et al., 2019a), between 22 and 39% of psychologists (Castelli Dransart et al., 2017), 55% of nurses (Takahashi et al., 2011) and 33% of social workers (Jacobson et al., 2004) will experience PUS during their training or career. Cerel et al. (2014) identified a “continuum of survivorship” after suicide, distinguishing those who are exposed to, affected by, or bereaved long-term or short-term by suicide. Considering PUS, all the members of the staff may encounter high levels of psychological distress and professional difficulties in the aftermath, including emotional, traumatic, and professional impacts (Séguin et al., 2014; Castelli Dransart et al., 2017; Leaune et al., 2019a, b). The emotional impact frequently includes shock, guilt, sadness, anger, failure, shame, and anxiety. Stress and traumatic reactions are also reported, including acute stress and posttraumatic stress disorders. Regarding professional impact, PUS may challenge the feelings of self-confidence and professional competence. A fear and avoidance of suicidal individuals, an impairment in professional decision-making or a decrease in work performance are notably reported. Some of the exposed professionals may also experience grief reactions or disenfranchised grief, i.e., a grief that is denied the right to exist because the impact of the loss is not recognized by peers, superiors or society (Doka, 1989). The majority of professionals will positively manage the impact of PUS through professional growth and encounter it as a learning and beneficial experience. In contrast, a minority of professionals will show high levels of impacts, with negative personal and professional outcomes. The term “second victims,” proposed to designate professionals exposed to adverse medical events in patients, has thus been used in the case of professionals impacted by PUS (Scott, 2019). The negative impact of PUS is significantly associated with a closeness to the deceased, a high level of the professional–client relationship and a lack of support and training. Castelli Dransart et al. (2015) notably reported that the lack of support in the aftermath was a risk factor for higher traumatic impact in the aftermath of PUS.

Postvention refers to the activities developed in the aftermath of a suicide to prevent negative health outcomes and facilitate recovery among the bereaved (Andriessen, 2009). Despite the growing evidence on the impact of PUS, the lack of institutional support in the aftermath has been widely documented (Ruskin et al., 2004; Castelli Dransart et al., 2015; Leaune et al., 2019a, b). Moreover, the literature on postvention programs dedicated to the support of health and social work professionals remains scarce. In retrospective studies, however, the participants have reported that support from peers, superiors or their institution can be both a protective factor and a predictor for adaptative coping strategies and lower levels of emotional, traumatic, and professional impacts (Castelli Dransart et al., 2015, 2017). Only one study qualitatively explored the effects of a half-day retreat dedicated to health and social work professionals previously exposed to PUS (Figueroa and Dalack, 2013). All respondents reported that participating in the retreat was a beneficial and helpful experience, highlighting the positive effects of the group intervention design. The effectiveness of this program to reduce the emotional, traumatic or professional impact of PUS was not assessed.

Hence, research dedicated to the development and evaluation of comprehensive and adaptive programs providing support to mental health professionals and social workers exposed to PUS is needed. In line with this urgent need for social change, this article aims to describe (1) the design and implementation of a postvention program (SUPPORT) for professionals exposed to PUS and (2) the protocol of the SUPPORT-S research-action study evaluating and improving the design of the SUPPORT program to effectively help professionals to cope with PUS.

## Method

### Development of the SUPPORT Program

The Center for Suicide Prevention (CSP) is an ambulatory care unit located in Lyon, France, that provides several types of suicide prevention activities, including brief contact interventions for people showing suicidal ideation or those who attempted suicide, outreach interventions for people showing suicidal behaviors, postvention counseling for individuals or family bereaved by suicide and information and training on suicide prevention.

**FIGURE 1:**
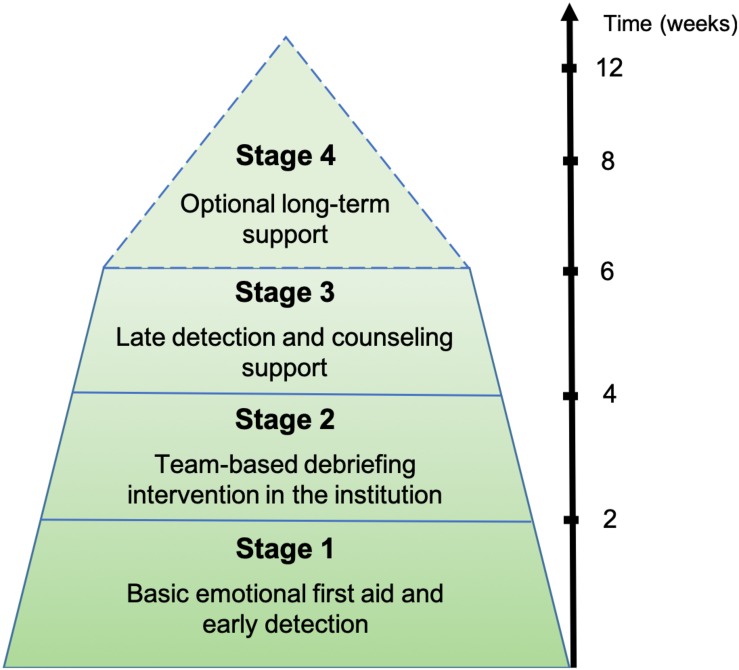
Schematic overview of the SUPPORT program.

According to the model of support for professionals after adverse events developed by Scott et al. (2010) our pluri-professional team of the CSP (psychiatrists, social and work psychologists, nurses, and psychiatric residents) designed a four-stage postvention program for mental health and social work teams exposed to PUS (SUPPORT, see Figure 1). The Scott three-tiered integrated model (STTIM) of interventional support after adverse event was developed by Scott et al. (2010) to provide an on-demand rapid intervention, ranging from immediate first aid support through peers and superior support to professional counseling for second victims. The STTIM relies on both local support from peers and superiors and supportive interventions by a trained team dedicated to the impact of adverse events on professionals.

Inspired and adapted from the STTIM, SUPPORT is a 6-week program divided into four distinct stages. The three following stages are mandatory: (a) basic emotional first aid at the local level; (b) team-based 1- to 2-h supportive intervention in the institution; and (c) follow-up, late detection. A fourth optional stage including long-term support and training or workshops on suicide prevention may be provided to professionals. The objectives of the SUPPORT program are to (1) buffer the traumatic, emotional and professional impacts in professionals, (2) improve the support perceived by exposed professionals, and 3) promote the return to normal functioning in the institution exposed to PUS.

#### Stage 1: Emotional First Aid

The first stage of the program includes early contacts by the CSP with the head of the exposed team (head of department, physicians, chief nurse, etc.) to provide them with an organizational framework of structured and synchronized crisis management. According to the guidelines for postvention interventions (Andriessen et al., 2019), the objective of this first stage is to build a crisis team dedicated to the management of the aftermath of PUS and the deployment of basic emotional first aid for the exposed professionals. The detection and initial support of highly impacted professionals (acute stress disorder, depressive reaction, suicidal ideation, etc.), the prevention of suicide contagion and the orientation to mental health care for those who are highly impacted are the most crucial issues of this first stage of the SUPPORT program. Individual consultations are notably proposed to the most strongly impacted professionals through a fast-track referral to the CSP. Stage 1 thus aims to promote basic emotional first aid and early detection for those who were the most impacted through local support from peers and superiors during the first days following a PUS, through the building of a crisis team.

#### Stage 2: Team-Based Intervention

The team-based intervention consists of a 1- to 2-h intervention in the institution where the suicide occurred, driven by two professionals (e.g., a psychiatrist or psychologist and a nurse) from the CSP in the month following a PUS. All of the exposed professionals are invited to participate in the intervention, during which a debriefing of the event and its impacts is performed. Exposed professionals are offered a supportive space to freely express their feelings about, emotions toward and experience of PUS. The intervention on the entire team aims to buffer the emotional, traumatic, and professional impacts, both in individuals and in the working team. In particular, the presence of the professionals who discovered the corpse or performed resuscitation techniques, those who were the closest of the deceased and those who were the most impacted is encouraged. Stage 2 thus aims to provide professional emotional aid at institutional level, through an in-depth team-based debriefing intervention.

#### Stage 3: Late Detection and Counseling

The third stage of the program seeks to perform late detection and counseling support for professionals who are strongly affected or traumatized by exposure to PUS. Those who feared attending the team-based intervention stage may notably receive individual debriefing during this stage. Stage 3 aims to promote the return to normal functioning in the institution, at both the individual and organizational levels. When stage 3 has not been effective in ensuring the recovery among professionals, a fourth stage can be added to properly support the impacted team.

#### Stage 4: Optional Long-Term Support

This optional stage is notably delivered if several suicides occurred or if the whole team is highly impacted by the suicide(s). The support of the management team and the close collaboration with the CSP are the most important components of this stage. A second debriefing intervention or a 3-hour suicide prevention training may be provided to the whole team. Workshops on the means and actions seeking to improve the prevention of suicidal behaviors or the support of professionals in the exposed setting are encouraged and organized, to involve impacted professionals in proactive and meaningful actions related to PUS. Stage 4 can last from one to three months and aims to provide in-depth long-term support for teams and institutions that have been strongly impacted by PUS.

### The SUPPORT-S Study

#### Study Objectives

##### Primary objective

The primary objective of the SUPPORT-S study is to improve the design of the SUPPORT program through the participatory involvement of professionals who deliver the intervention and those who receive it.

##### Secondary objectives

The SUPPORT-S study aims to evaluate the effectiveness of the SUPPORT program to buffer the emotional, traumatic and professional impacts and to improve the perceived social support for professionals exposed to PUS. The SUPPORT-S study also seeks to provide more insights into the consequence of PUS on both professionals and the organization and to identify markers that may help in implementing programs adapted to different professional settings.

#### Study Setting

The SUPPORT-S study will take place in Rhône, France, which is the geographical area of intervention for the CSP. The SUPPORT program is designed to be implemented in the following institutions: psychiatric hospitals, general hospitals, nursing homes and social work institutions. The study will start in February 2020 and last 18 months. Every psychiatric and general hospitals, nursing homes and social work institutions of the geographical area will receive in February 2020 detailed information on the SUPPORT-S study. A mail and an email will be sent to all executive managers.

#### Inclusion and Exclusion Criteria

Every professional working in the institution in which PUS occurred are eligible for participation in the SUPPORT-S study, without any exclusion criteria. Only professionals who do not agree to participate will not be included in the study but they will receive the postvention intervention if they wish to. The eligible professionals will be informed of the concepts, design and running of the study through an information sheet and oral explanations at inclusion.

#### Study Design

**FIGURE 2:**
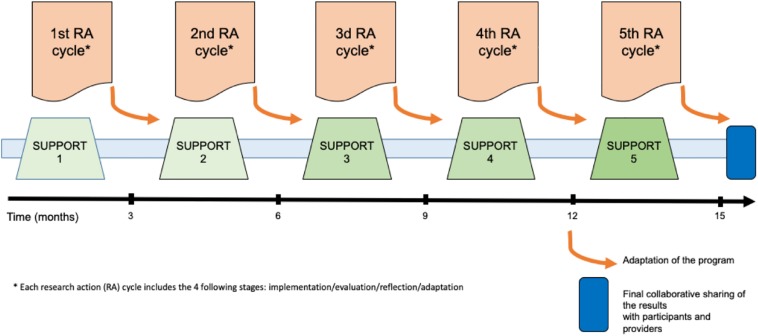
Schematic overview of the SUPPORT-S study.

SUPPORT-S is a mixed method collaborative and participatory action research study, including a total of five SUPPORT interventions over a total of 15 months (Figure 2). SUPPORT-S seeks to evaluate both the effectiveness of the program from the perspective of professionals who receive the intervention (i.e., participants) and those who deliver it (i.e., providers) through an iterative participatory process.

##### Action research

Action research is an approach that involves active collaboration *in situ* between researchers and participants to develop a process through knowledge building and social change (Lewin, 1946). Action research gives credence to the development of powers of reflective thought, discussion, decision, action, evaluation and revision by people or professionals participating in a collective research, through active participation during iterative sequences of those who have to carry out the work in the exploration of problems that they encounter (Adelman, 1993). The affective and cognitive involvement of participants is critical for the production of empirical knowledge and social modifications in professional practices. Cordeiro and Soares (2018) identified the following four principles of action research: (1) participation and collaboration; (2) a cycle of implementation, action, reflection and adjustment; (3) knowledge building that considers participants’ realities; and (4) social change and problem solving. These principles will be assumed in the SUPPORT-S study through the cycling process of local implementation/evaluation/reflection/adaptation and the active participation of professionals in the evaluation process through restitutions of the results and the conditions of the implementation. The action research process will be held through the following three main activities: (1) the collaborative sharing of results for all professionals after each SUPPORT intervention, (2) the cycling process for each SUPPORT intervention, and (3) the final collaborative sharing of results at the end of the study.

The *collaborative and participatory approach* will notably rely on the collaborative sharing of results, during which professionals (i.e., participants and providers) will be allowed to express their feelings about and experience with the SUPPORT program to propose adjustments for the program. The discussion, revision and validation of the results by the participants ensure their social relevance for the modification of practices in the institution and the implementation of new professional postures in professionals. This approach thus has a transformative role in the pluri-professional collective workforce rather than only focusing on individuals by involving them as active actors and not only participants of the research (Durif-Bruckert and Gonin, 2011; Budig et al., 2018). Moreover, the collaborative approach is based on the implications of social sciences researchers and clinical researchers who are also providers of the program. According to the notion of the “expanded scientific community” developed by Oddone et al. (1981), our participatory research action design thus involves a pluri-professional collaboration between participants, providers and researchers. This concept was developed to produce in-depth and empirically grounded knowledge on work-related psychosocial risk prevention and emphasizes the importance of the social context to improve efficacy and well-being in professionals. The lack of institutional support reported by professionals in the aftermath of PUS indicates the need to drive a substantial social change in institutional practices regarding postvention issues. The research action design of the study thus aims to allow the dissemination of a comprehensive and adaptative intervention among different institutions and professional settings. The collaborative and participatory approach of the SUPPORT-S study is understood at the same time as a way to gain effectiveness in the intervention, as a way to limit the biases related to mixed method design, and as an ethical posture in the research process and the interpretation of the results.

The *cycling process* of the SUPPORT-S study will be one of the key components of the action research design. Indeed, the local implementation of the SUPPORT program after a PUS will be performed throughout the following repeated process: (a) implementation, (b) qualitative and quantitative evaluation, (c) collaborative sharing of the results, and (d) adjustment of the program according to the restitution and evaluation (Table 1). Notably, the reflection on and modification of the SUPPORT program after each local implementation will ensure the cycling process of implementation/evaluation/reflection/adaptation through the active participation of both providers and participants. For each local implementation, the sequence will be repeated, and the program will be progressively modified, revised and adapted according to the cycling process. The revision and adaptation of the SUPPORT program after each implementation, through the active participation of providers and participants, will improve its effectiveness and relevance to help professionals in coping with PUS. Moreover, the cycling process is a methodological means to ensure the collaborative and participatory approach of the study, as providers and professionals are proactively involved in the research process.

**TABLE 1:** The cycling process of the SUPPORT-S study.

Stage of the cycling process	Actions
Implementation	The CSP is informed of a PUS through emails or phone calls by the chief department of the institution where it occurred. Through a partnership developed with the Regional Health Agency, the CSP can be informed of every PUS occurring in nursing homes or social work organizations in the area and contact the chief department. After oral and written information of all exposed professionals in the institution, the SUPPORT program is implemented in the setting
Evaluation	• Collection of qualitative data: ethnographic observation, five individual semidirected interviews with participants, activity analysis with the SUPPORT program providers • Collection of quantitative data: traumatic impact (IES-R), emotional impact (DES-IV), professional impact (non-validated scale) and perceived support (P3SP) among participants/exposed professionals • Merging of the qualitative and quantitative data • Data analysis and report
Collaborative sharing of the results	Discussion, revision and validation of the results between researchers, providers and participants (workshop). Participants and providers freely express their feelings about and experience with the SUPPORT program to propose an adaptation of the program
Adaptation	Adaptation of the SUPPORT program (content, form) according to the revisions proposed during the collaborative sharing of data, before the next implementation

*CSP, Center for Suicide Prevention; PUS, Patient or user suicide; IES-R, Impact of Event Scale – Revised; DES-IV, Differential Emotions Scale – IV; P3SP, Perceived Social Support Scale adapted for Professionals.*

##### Mixed method evaluation

Mixed method studies have been shown to be effective in evaluating the implementation of new prevention programs in mental health (Palinkas et al., 2011). Mixed methods design focuses on collecting, analyzing and merging both quantitative and qualitative data into one study. According to the taxonomy of mixed method studies described by Palinkas et al. (2011), the structure of the SUPPORT-S study relies on a simultaneous collection of qualitative and quantitative data. Regarding the function, the two datasets will be collected in complementarity, e.g., qualitative data will be used to provide depth of understanding, and quantitative data will be used to provide breadth of understanding. The process of data analysis will be performed by merging the two datasets and actually bringing them together. The SUPPORT-S study is thus designed as a simultaneous complementary merging mixed method study.

#### Data Collection

**FIGURE 3:**
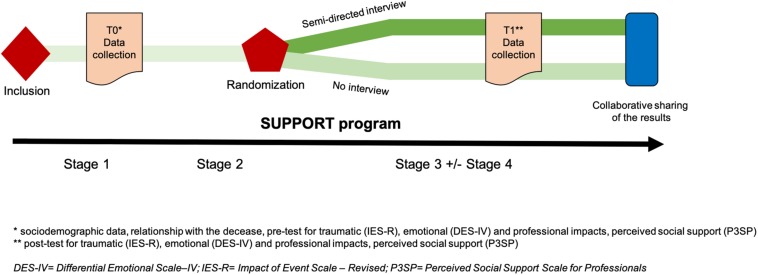
Participants’ trajectories in the SUPPORT-S study.

According to the mixed method research design of the study, quantitative and qualitative data will be simultaneously and complementarily collected to obtain an in-depth evaluation of the implementation and effectiveness of the SUPPORT program (Figure 3).

##### Quantitative data

A collection of individual quantitative data will be performed before (T0) and after (T1) the SUPPORT program among professionals exposed to a PUS. Sociodemographic data and information on the therapeutic relationship with the deceased will be collected at T0. A pre/post measure of emotional, traumatic and professional impacts and perceived social support will be performed at T0 and T1.

The following sociodemographic characteristics of participants will be collected at T0: age, gender, profession, and years spent working in the organization.

The following information on the relationship between the professional and the event or the patient or user who died by suicide will be collected at T0: connection with the deceased, discovery of the corpse, resuscitation techniques used, being at work or not the day of the event, and therapeutic alliance with the deceased.

The traumatic impact will be assessed at T0 and T1 through the French version of the *Impact of Event Scale-Revised* (IES-R; Horowitz et al., 1979; Weiss and Marmar, 1997; Brunet et al., 2003). The IES-R is a 22-item self-report questionnaire assessing intrusion, avoidance and hyperarousal symptoms in the previous seven days before completion. Each item is rated on a 5-point Likert scale ranging from 0 (not at all) to 4 (extremely), for a total score of 88. According to previous literature, we will retain a cut-off score of 24/88 to indicate a significant traumatic reaction (Bienvenu et al., 2013) and a cut-off of 34/88 for PTSD (Asukai et al., 2002). The French version of the IES-R shows a good internal consistency (alpha coefficients ranging from 0.81 to 0.93) and satisfactory test-retest reliability (correlations coefficients ranging from 0.71 to 0.76) (Brunet et al., 2003).

The emotional impact will be obtained at T0 and T1 through the French version of the *Differential Emotions Scale IV* (DES-IV; Ricard-St-Aubin et al., 2010). The DES-IV is a 36-item questionnaire assessing the level of expression of the twelve following emotions: interest, joy, surprise, anger, contempt, disgust, sadness, fear, guilt, shame, shyness, and self-hostility. The emotions are measured through a 6-item Likert scale ranging from 1 (rarely or never) to 5 (very often), for a total score ranging from 36 to 180. The DES-IV shows good temporal stability (correlations coefficients ranging from 0.49 to 0.79) (Ricard-St-Aubin et al., 2010).

The professional impact will be assessed at T0 and T1 through a non-validated 18-item questionnaire built by our pluri-professional team and adapted from non-validated questionnaires on self-efficacy at work (self-confidence, problem-solving, and decision-making) and modifications in practice. The participants will be asked through close-ended questions about the self-perception of their effectiveness at work, the perception of modification in their professional practice and the type of modification that they perceive. Regarding the self-reported modifications in professional practice, a fear and avoidance of suicidal patients, an increased tendency to hospitalize patients, a fear of granting passes for patients and a prolonged duration of hospitalization are considered negative professional impacts. A better assessment of suicidal ideation in patients, an increased tendency to ask advice to colleagues, a better trackability of information in patients’ files and an interest in suicide prevention are considered positive professional impacts. Regarding self-efficacy, the perceived loss of self-confidence or self-control and perceived difficulties in problem solving or decision-making will be considered as negative professional impacts. The presence of at least three items indicating a negative impact on professional practice or self-efficacy will be considered as a negative professional impact.

Perceived support will be assessed through the *Perceived Social Support Scale for Professionals* (P3SP; Collange et al., 2016). The P3SP is a 12-item questionnaire evaluating the following four types of perceived social support provided by peers, superiors or the institution in the context of the workplace: (1) instrumental support (e.g., material support, technical assistance), (2) emotional support (e.g., being listened to), (3) informational support (e.g., advice, information, counseling), and (4) esteem support (e.g., positive feedback, constructive appreciation). The satisfaction regarding the four types of perceived support is assessed through a 4-item Likert scale ranging from 0 (not satisfied) to 3 (very satisfied), for a total score ranging from 0 to 36. The P3SP shows good sensitivity and specificity (Collange et al., 2016). Three scores are especially measured by the P3SP questionnaire: (1) level of perceived support from peers and collaborators (from 0 to 12), (2) level of perceived support from the institution (from 0 to 12), and (3) level of satisfaction of perceived support from superiors (from 0 to 12). The P3SP shows very good internal consistency (Cronbach’s alpha ranging from 0.82 to 0.93) (Collange et al., 2016).

##### Qualitative data

An *ethnographic observation* of the local implementation of the SUPPORT program will be performed by social sciences researchers (CD-B, MV, and BCu) to collect in-depth observational data on the implementation of the program. The observation will notably focus on the reaction of the professionals exposed to PUS, and how they manage and experience the implementation of the SUPPORT program. The interactional processes between professionals delivering the program will also be examined.

For each intervention, five exposed professionals will be randomly selected to participate in an *individual semistructured interview* at the end of the program. The semistructured interviews will last between 30 and 60 min and will be performed by one or two social researchers (CD-B, MV, and BCu) in the workplace of participants. The participants will be interviewed regarding their individual experience of the SUPPORT program, including: how they perceived the program for themselves and for their team, which mechanisms led to its effectiveness and how the program could be improved. The content of the interview will be recorded and anonymized.

An *activity analysis* will be performed with the providers of the intervention. An activity analysis is an effective means to analyze complex processes occurring in professional practices (e.g., professionalization processes, interactions between professionals, internalization of professional identities, etc.) (Bedny and Karwowski, 2004). We will use the methodology of auto- and allo-confrontation (Mollo and Falzon, 2004). Regarding methodological issues, in auto-confrontation, subjects are confronted with their own activity, whereas in allo-confrontation they are confronted with an activity that they practice but which is performed by someone else. Regarding purpose issues, auto-confrontation aims to reveal the cognitive processes underlying the activity, whereas allo-confrontation allows subjects to develop their knowledge by becoming aware of other types of representations (Mollo and Falzon, 2004). To perform the activity analysis, the providers of the program will be filmed during their intervention in the institution (stage 2 of the SUPPORT program). In auto-confrontations, they will be asked to comment on and to express their experience with and feelings about moments of their intervention. In allo-confrontation, the comments will be made by other providers from the CSP to enrich the discussion about professional practices and question the different ways of running the intervention.

##### Estimated number of participants

The inclusion of 150 exposed professionals is expected for the collection of quantitative data. Considering the five local implementations of the SUPPORT program throughout the SUPPORT-S study, the inclusion of 150 exposed professional is needed to observe a 30% decrease in the mean IES-R score between T0 and T1 with a 90% statistical power.

Of them, a total of 25 professionals will participate in the semistructured interviews. According to previous qualitative studies on professionals’ experiences of adverse events in which similar (Ullström et al., 2014; Ferrús et al., 2016) or lower (Chan et al., 2018; Kable et al., 2018) samples were included, the inclusion of 25 professionals in semidirected interviews should provide sufficient saturation for the representativeness and reliability of the data. However, the sample of participants in semidirected interviews could be increased if saturation is not reached.

#### Data Analysis

The quantitative and qualitative data collected in the study will be separately analyzed through independent statistical and qualitative analyses.

##### Quantitative analysis

Data manipulation and analyses will be performed using R software (R 3.4.1). Qualitative variables will be summarized using numbers and percentages, and quantitative variables will be described using either means and standard deviations or medians and interquartiles. Quantitative variables will be analyzed with the appropriate test, depending on the application conditions. The evolution between T0 and T1 of the following four dependent variables will be measured: (1) PTSD reaction (IES-R > 24), (2) high emotional impact (DES-IV > 108), (3) negative professional impact (e.g., the presence of at least three items indicating negative impact on professional practice or self-efficacy), and 4) low perceived social support (P3SP < 12). For each variable, the total scores will be compared between T0 and T1 using paired Student’s *t*-test or non-parametric sum rank test for matched samples, depending on the distribution of the variables. Then, the proportion of subjects above the cut-off scores will be compared between T0 and T1 using the McNemar test. For the main outcome criteria analysis (IES-R score) a multivariate analysis will be conducted using a multivariate linear mixed model to adjust for main confounders: age, gender, profession, relationship with the decease. All tests will be two-tailed and the statistical significance threshold will be set at 5%.

##### Qualitative analysis

Based on the narratives of the semidirective interviews, a content analysis will be performed, with several chronological phases (reanalysis, operation of equipment, and interpretation) (Bardin, 2013). According to a new method developed by Renz et al. (2018), two data analyses (i.e., manual and computer based) will be combined to enhance the trustworthiness of the results. The first method is based on a manual content analysis (Graneheim et al., 2017). Three authors (EL, BCu, and CD-B) will first look at the apparent messages through a repeated reading of the transcripts to achieve immersion and obtain a sense of the whole. In addition, this first reading will allow us to define thematic and formal categories relevant for later coding speeches. Units of meaning will then be independently identified, categorized and put into relation to identify axes of transversal meanings. This process will allow us to classify the elements and to emit a simplified representation of the raw data. The second method will use a computer-based content analysis, through the NVivo software, and will be performed by another author (MV). NVivo is a computer-assisted qualitative data analysis software that allows for qualitative inquiry beyond the coding, sorting and retrieval of data (Wong, 2008). The benefits of using NVivo are outlined in terms of facilitating teams of researchers to systematically and rigorously synthesize qualitative data.

Finally, the results issued from the two methods will be grouped and organized through a coding scheme.

### Funding Sources and Ethical Approval

The SUPPORT-S study is funded by the Scientific Research Committee from the Centre Hospitalier le Vinatier and University Lyon 2. The study received ethical approval from the Ethical Review Board Sud-Est IV of Clermont-Ferrand (registration number 2019/CE67).

## Expected Results

Although the impact of PUS on mental health professionals and social workers has been well described, the SUPPORT-S study will be the first to evaluate the effectiveness of a postvention program for professionals exposed to PUS. The mixed method and action research design will allow us to collect both in-depth qualitative and quantitative data on the effectiveness of the implementation of the SUPPORT program. The multidisciplinary approach through the collaboration of researchers and clinicians from different disciplines and theoretical backgrounds is another strength of the study, as it will improve the strength and generalizability of our conclusions. While previous studies have relied on retrospective data collection through large surveys or qualitative interviews (e.g., individual interviews or focus-groups), the longitudinal design of the current study will offer new insights and perspectives on PUS and its impact on professionals and institutions.

Our study protocol has several limitations. First, the non-controlled design prevents us from making definitive inferences on the effectiveness of the program. The use of a research-action approach may also limit the representativeness of the sample, induce selection biases and limit the ability to avoid misinterpretation in the researchers’ conclusions. Second, the SUPPORT-S study is a monocentric study performed in the French context. This setting may impede the generalizability of our conclusions and results, due to the particularities of the French health system and social policies. Finally, the small number of local implementations (5) may limit the power of our results and conclusions. However, the following four main bias limitations will be used in the SUPPORT-S study: (a) triangulation, (b) randomization, (c) saturation, and (d) participatory approach. *Triangulation* refers to the use of multiple methods or data sources in qualitative research to develop a comprehensive understanding of phenomena (Patton, 1999; Carter et al., 2014). Denzin (1978) and Patton (1999) identified the following four types of triangulation: (a) method triangulation, (b) investigator triangulation, (c) theory triangulation, and (d) data source triangulation. The four types of triangulation will be ensured in the SUPPORT-S study through the mixed method and action research design (method and data source triangulation) and the involvement of researchers from different disciplines and theoretical backgrounds (theory and investigator triangulation). An intramethod triangulation will also be added through the two methods of content analysis previously described in the paragraph on qualitative analysis. The *randomization* of professionals to participate in individual semidirected interviews will limit the selection bias by randomly ensuring the representativeness of the sample and avoiding the biased selection of, for instance, more motivated professionals. *Saturation* is used in qualitative designs as a criterion for discontinuing data collection and analysis (Saunders et al., 2018). According to the taxonomy developed by Saunders et al. (2018), the following four types of saturation are defined: (a) theoretical saturation (i.e., the development of theoretical categories), (b) inductive thematic saturation (i.e., the emergence of new codes or themes), (c) *a priori* thematic saturation (i.e., the degree to which identified codes or themes are exemplified in the data), and (d) data saturation (i.e., the degree to which new data repeat what was expressed in previously collected data). The saturation used in the SUPPORT-S study will focus on the data saturation and the inductive thematic saturation. The *participatory approach* is an effective means to limit the misinterpretation of the collected data by the researchers. The involvement of participants in the restitution process promotes the ability to adjust the program as well as the conclusions issued from the research through their direct comments of the results.

## Conclusion

Health and social work professionals are frequently exposed to PUS and may encounter several ranges of emotional, traumatic or professional impacts in the aftermath. The lack of support in the aftermath of PUS has been reported by a high proportion of exposed professionals, indicating the urgent need to implement and evaluate innovative programs dedicated to facilitating recovery and preventing negative health outcomes after a PUS. The SUPPORT program is a postvention program designed to provide comprehensive, adaptative and effective support to professionals impacted by a PUS. The SUPPORT-S study is a mixed method collaborative and participatory action research project aiming to (1) improve the design of the SUPPORT program, (2) evaluate the effectiveness of the SUPPORT program to buffer the emotional, traumatic and professional impacts and to improve the perceived social support for professionals exposed to PUS and (3) provide more insights into the consequences of PUS on both professionals and organizations. The simultaneous and complementary collection and analysis of qualitative and quantitative data will offer an in-depth evaluation of the program’s implementation and effectiveness. The action research design will also improve the limitation of biases and improve the generalizability of the conclusions. The results of the study will allow us to disseminate an effective postvention program for professionals and institutions encountering PUS.

## Ethics Statement

The studies involving human participants were reviewed and approved by Comité de Protection des Personnes Sud-Est VI. The patients/participants provided their written informed consent to participate in this study.

## Author Contributions

EL, MP-T, NC, BCh, and EP contributed to the conception and design of the SUPPORT program. EL, BCu, MV, NC, A-FP, JH, and CD-B contributed to the conception and design of the study. EL wrote the first draft of the manuscript. BCu, AF, JH, and CD-B wrote the sections of the manuscript. All authors contributed to manuscript revision, read, and approved the submitted version.

## Conflict of Interest

The authors declare that the research was conducted in the absence of any commercial or financial relationships that could be construed as a potential conflict of interest.
